# Perceiving animacy from kinematics: visual specification of life-likeness in simple geometric patterns

**DOI:** 10.3389/fpsyg.2023.1167809

**Published:** 2023-06-02

**Authors:** Giulia Parovel

**Affiliations:** Department of Social, Political and Cognitive Sciences, University of Siena, Siena, Italy

**Keywords:** animacy, intentionality, perception of causality, perceptual life-detector, life-like kinematics, expressive qualities, Michotte, Heider and Simmel

## Abstract

Since the seminal work of Heider and Simmel, and Michotte’s research, many studies have shown that, under appropriate conditions, displays of simple geometric shapes elicit rich and vivid impressions of animacy and intentionality. The main purpose of this review is to emphasize the close relationship between kinematics and perceived animacy by showing which specific motion cues and spatiotemporal patterns automatically trigger visual perceptions of animacy and intentionality. The animacy phenomenon has been demonstrated to be rather fast, automatic, irresistible, and highly stimulus-driven. Moreover, there is growing evidence that animacy attributions, although usually associated with higher-level cognition and long-term memory, may reflect highly specialized visual processes that have evolved to support adaptive behaviors critical for survival. The hypothesis of a life-detector hardwired in the perceptual system is also supported by recent studies in early development and animal cognition, as well as by the issue of the “irresistibility” criterion, i.e., the persistence of animacy perception in adulthood even in the face of conflicting background knowledge. Finally, further support for the hypothesis that animacy is processed in the earliest stages of vision comes from recent experimental evidence on the interaction of animacy with other visual processes, such as visuomotor performance, visual memory, and speed estimation. Summarizing, the ability to detect animacy in all its nuances may be related to the visual system’s sensitivity to those changes in kinematics – considered as a multifactorial relational system - that are associated with the presence of living beings, as opposed to the natural, inert behavior of physically constrained, form-invariant objects, or even mutually independent moving agents. This broad predisposition would allow the observer not only to identify the presence of animates and to distinguish them from inanimate, but also to quickly grasp their psychological, emotional, and social characteristics.

## Introduction

In a fairly calm place, a sudden impression of motion immediately attracts our curiosity and awakens in us the impulse to discover the nature of that movement, to see if the moving object is an animated being, e.g., a cat or a fly, or a casual displacement of an inanimate object, e.g., a falling leaf. Motion is one of the key characteristics of animate things and thus, for our ancestors, immediately seeing and reacting to a potential danger or prey certainly played a vital role for survival. Detecting the presence of a living organism solely on the basis of its movement, without having to examine further visual details, can bring a relevant advantage for instance when the visibility conditions are lacking, because the moving object is too far away or blurred, shaded, partially hidden or camouflaged.

The possibility of manipulating the kinematic variables independently from other visual characteristics of the moving objects, and of exploring the relationship between these variables and the meaningful information that emerges, is precisely one of the reasons why the study of *animacy* – i.e., the character of “being alive” – is fascinating for vision scientists. Since the seminal works by Heider and Simmel (1944) and Michotte (1946/1963) to more recent research, many studies demonstrated that, under appropriate conditions, even displays of simple geometrical shapes might give rise to rich and vivid impressions of animacy and intentionality, by virtue of their simple movements and interactions. The main purpose of the present review is to highlight the close relationship between visual kinematics and perceived animacy, by drawing the whole picture of the specific motion cues and spatiotemporal patterns which automatically trigger rich and vivid visual percepts of animacy in geometrical patterns, independently from other appearance-based visual cues. A growing body of evidence for this specific sensitivity to kinematics-based animacy, as we will see, comes from a variety of research fields, ranging from experimental psychophysics to developmental psychology and causal reasoning, as well as animal cognition and neuroscience. It involves different methodologies and theoretical assumptions that are not always easy to compare. The paper then turns to the debate about the processes underlying animacy, contrasting the two main theoretical positions present in the literature: one, that the observer must activate representations – or schemas – and expectations in long-term memory in order to recognize the ongoing events (see Rips, 2011), and the other suggesting that observer can directly perceive high-level properties such as animacy phenomena, which are fairly fast, automatic, irresistible, and highly stimulus-driven (Scholl and Tremoulet, 2000; Scholl and Gao, 2013). The following sections review the increasing empirical evidence that animacy and its related properties – such as intentionality or social causality – are hardwired into the brain (Rutherford, 2013; Hafri and Firestone, 2021; Lemaire and Vallortigara, 2023). In particular, we examine recent findings on sensitivity to kinematic-based animacy cues in newborns; discuss the issue of “irresistibility,” i.e., the persistence of animacy perception into adulthood even in the face of conflicting background knowledge and the co-presence of incongruent visual information; and review recent experimental evidence on how animacy interacts with other visual processes, such as visuomotor performance, visual memory, and speed estimation. The final section attempts to synthesize into a unified framework the features that single moving objects – as well as more complex kinematic patterns – must exhibit in order to trigger the animacy response of a hypothetical “life detector” (Vallortigara, 2012). In particular, it takes into account the visual system’s predisposition to perceive spatiotemporal relationships between movements that are intrinsically endowed with information about the nature of ongoing events (Hafri and Firestone, 2021).

## Animacy by simple kinetic variations

As observed since Aristotle, a salient property of animated motion is its active character, quite different from the passive motion of a falling body or clouds driven across sky.

“Take, for instance, any animal: the animal moves itself, and we call every movement natural, the principle of which is internal to the body in motion” (Aristotle, Physics, vol. V, p. 307).

Indeed, self-propulsion is considered a strong cue to animacy in experimental psychology. The first systematic exploration of the role of *self-propelled* motion in animacy was carried out by the Belgian psychologist Michotte (1946/1963) in his research into animal locomotion. Living movements, he wrote, “have the appearance of being activities of which the object itself seems to be *the source*.” He presented to observers non-rigid extending and contracting rectangles moving like a caterpillar or swimming like a frog (see a demonstration at: https://youtu.be/glEPmTd_EtA). Michotte reported that subjects showed great surprise and, without any prompting, literally described an animal crawling or creeping – an object *which moves of its own accord* (Michotte, 1946/1963, p. 185).

Recent research in this field confirmed the observation that a powerful perceptual cue to convey animacy is to appear self-propelled, i.e., moving by itself in the absence of an external cause, thus implying evidence of an inner energy source. Based on this evidence, many scholars have theorized about the involvement of causal attribution inferences and specialized cognitive processes in animacy recognition (Stewart, 1982; Leslie, 1984; Dasser et al., 1989; Premack, 1990; Scholl and Tremoulet, 2000; Gergely and Csibra, 2003; Csibra, 2008). Developmental studies suggested that infants recognize and distinguish self-moving objects from inert ones as early as 6 or 7 months of age (Leslie and Keeble,1987; Woodward, 1998; Pauen and Träuble, 2009). Di Giorgio et al. (2016) showed that seeing the onset of the self-propelled motion of an object, in contrast to it emerging from behind an occluding rectangle, is a crucial visual cue underlying animacy perception that allows even human newborns to differentiate between self- and non-self-propelled objects. When the onset of the motion is removed, newborns do not manifest any visual preference. Not only humans, but also newly hatched chicks demonstrated having an innate sensitivity to self-produced motion (Mascalzoni et al., 2010). Both these last works support the hypothesis of the presence already at birth of a predisposition to detect specific visual motion cues that might be a precursor to animacy percepts (Di Giorgio et al., 2016; Lemaire and Vallortigara, 2023).

Strictly related to the manifestation of an internal driving force in self-propelled objects is the prerogative of animate beings to not dissipate or even increase their observable kinetic energy. More in general, according to the Newtonian principles violation hypothesis, animacy can be triggered by simple movements that violate energy conservation (e.g., Stewart, 1982; Bingham et al., 1995; Gelman et al., 1995). Moving objects with sudden changes in direction and speed are more likely to appear animated (Tremoulet and Feldman, 2000; Träuble et al., 2014; van Buren et al., 2016).

Recently an animacy pattern has been revealed also in bouncing-like scenarios, obtained with a disk moving vertically downwards and then upwards, one or more times (Parovel et al., 2022; Vicovaro et al., 2023; see here few samples of the stimuli: https://youtu.be/Dt-QyXAjqNk). In specific conditions, depending on the simulated value of the coefficient of restitution, the visual impression of physical bouncing gave way to animate *jumping*. The most compelling animated jumping-like motions tended to occur when the stimuli showed a clear violation of energy conservation as well as multiple bouncing cycles (Bingham et al., 1995; Parovel et al., 2022; Vicovaro et al., 2023).

Speed itself seems to be a relevant factor for inducing animacy; Szego and Rutherford (2007) examined objects moving at constant speed, without changes in trajectory, and revealed that relatively faster objects appear animate, even if the speed difference is illusory. The authors argued that an inanimate object traveling across such a surface would be slowed by friction, so any object able to maintain a constant speed across a surface was likely to be self-propelled.

Also, Szego and Rutherford (2008) found that the visual system is sensitive to changes in the orientation of stimuli relative to gravity. By comparing “rising” vs. “falling” dots moving at the same speed, dots in upward motion were judged as animate more often than those moving downwards.

Consistently, it has been shown that observers are much more sensitive to speed changes in the direction opposite to the direction of gravity when they are required to report whether or not a speed change has occurred (Nguyen and van Buren, 2023).

Regarding the shape of the trajectory, other cues of living motion have been identified: (a) in C-shaped or S-shaped paths (Stewart, 1984; Gelman et al., 1995; Blythe et al., 1996, 1999); (b) in the mimicking of natural stimuli, such as flies, through speed and direction changes (Schultz and Bülthoff, 2013); furthermore, (c) in the alignment of the major axis of the shape of the moving object to its trajectory (Tremoulet and Feldman, 2000). A preference for parallelism between the principal axis of a moving object and its trajectory was observed even in visually-naive newborn chicks (Rosa-Salva et al., 2018).

Other decisive factors for intensifying the perception of animacy are the interactions of movements with other entities – discussed in depth in the next sections – and *goal-directedness*. Opfer (2002) for example demonstrated that unfamiliar shapes, i.e., blobs, with similar trajectories are identified by children and adults as living organisms if their movements are goal-directed (see also Gergely and Csibra, 2003; Schultz et al., 2005; Csibra, 2008). Even in displays where there was no visible goal, subjects often described the self-propelled movements as goal-directed but toward a target outside the observer’s visible range, conveying an impression of intentionality (Tremoulet and Feldman, 2000, p. 947).

## Perceiving the relationship between movements: intentions and emotions

As discussed above, the kinematic properties of a single moving object can generate an impression of animacy. However, according to some authors, autonomous motion in itself cannot be a decisive factor in distinguishing animate from inanimate events, because it can be an ambiguous source of information (Gelman et al., 1995; Opfer, 2002). Indeed, a large amount of research has shown how interactions between simple moving shapes can elicit more sophisticated attributions of intentionality and psychological or emotional states, therefore supporting the fundamental role that perception plays in social cognition. Of course, as we will see, phenomenological reports, while essential to attest the emergence of these properties, must be supported by less direct methods of investigation to demonstrate the involvement of genuine visual processing in these scenarios.

**Figure 1:**
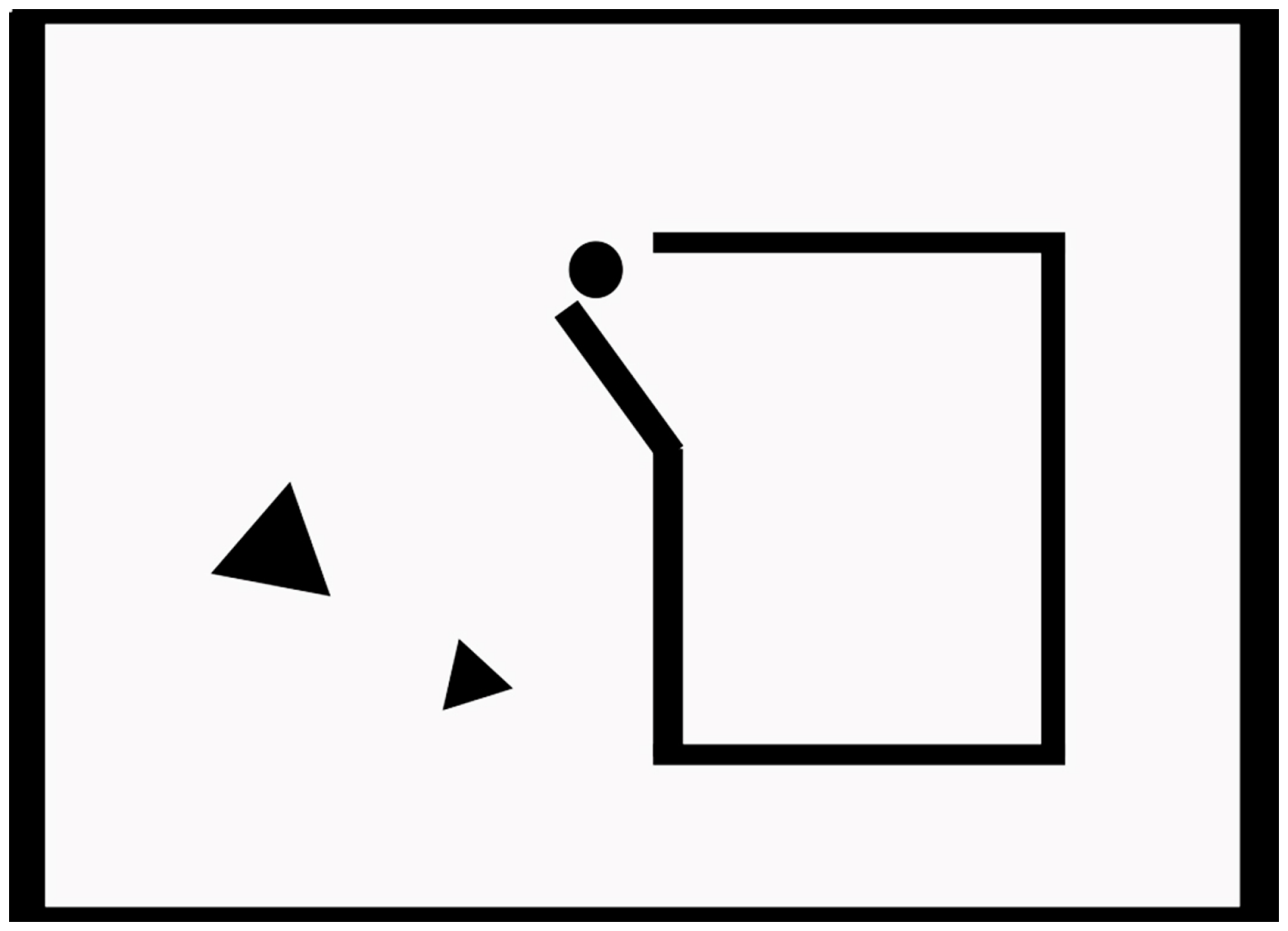
A frame of the classic Heider and Simmel (1944) animation.

In the classic Heider and Simmel (1944) experiment, observers presented with a cartoon-like animation in which two triangles and a disk interacted in and around a rectangular shape, attributed emotions, psychological traits, and intentions to those shapes. They used adjectives such as aggressive, shy, brave, intimidating, chasing, escaping, etc. and described the sequence as an interpersonal story in a remarkably consistent way. From the entire animation the authors extrapolated four basic combinations of movements: *successive* movements with or without spatial contiguity (corresponding for example to launching or joined movements in which one “causes” the other, such as action-at-a-distance), and *simultaneous* movements with or without spatial contact (corresponding to pushing, attracting, chasing and similar events). The movements, conjectured the authors, appeared organized in terms of acts of persons, and the interpretation of these movement-combinations varied according to the unit seen as the causal origin (the original animation is available at: https://youtu.be/8o6d9mUXwtg) (Figures 1).

According to Michotte (1950/1991), the relationship occurring between two or more moving objects within specific kinetic structures gives rise to primitive phenomena. These are to be considered quite different from the meanings which – under the influence of past experience – are attached to simple impressions of motions, merely juxtaposed in space and time. Certain combinations of visual stimuli, defined as to their distance, their speed, etc., cause certain specific impressions, for example, the impression “that an object A goes *toward* an object B,” “that A *pursues* B,” “that A *bumps* B,” “that A *chases* or *repels* B,” “that A *goes to find* B and *take it away*,” and so on (Thinès et al., 2014, p. 104). These phenomena, stated Michotte, depend essentially on the system of stimulation, so that every notable modification in this system brings about a change in the expressed meaning of the relation. Within a certain distance between the objects, for instance, the impression of “approaching” is much stronger than the simple “shortening of the distance,” and can vary in several qualitative ways such as a “friendly” or “aggressive” approach. On the contrary, speed varies only quantitatively.

**Figure 2:**
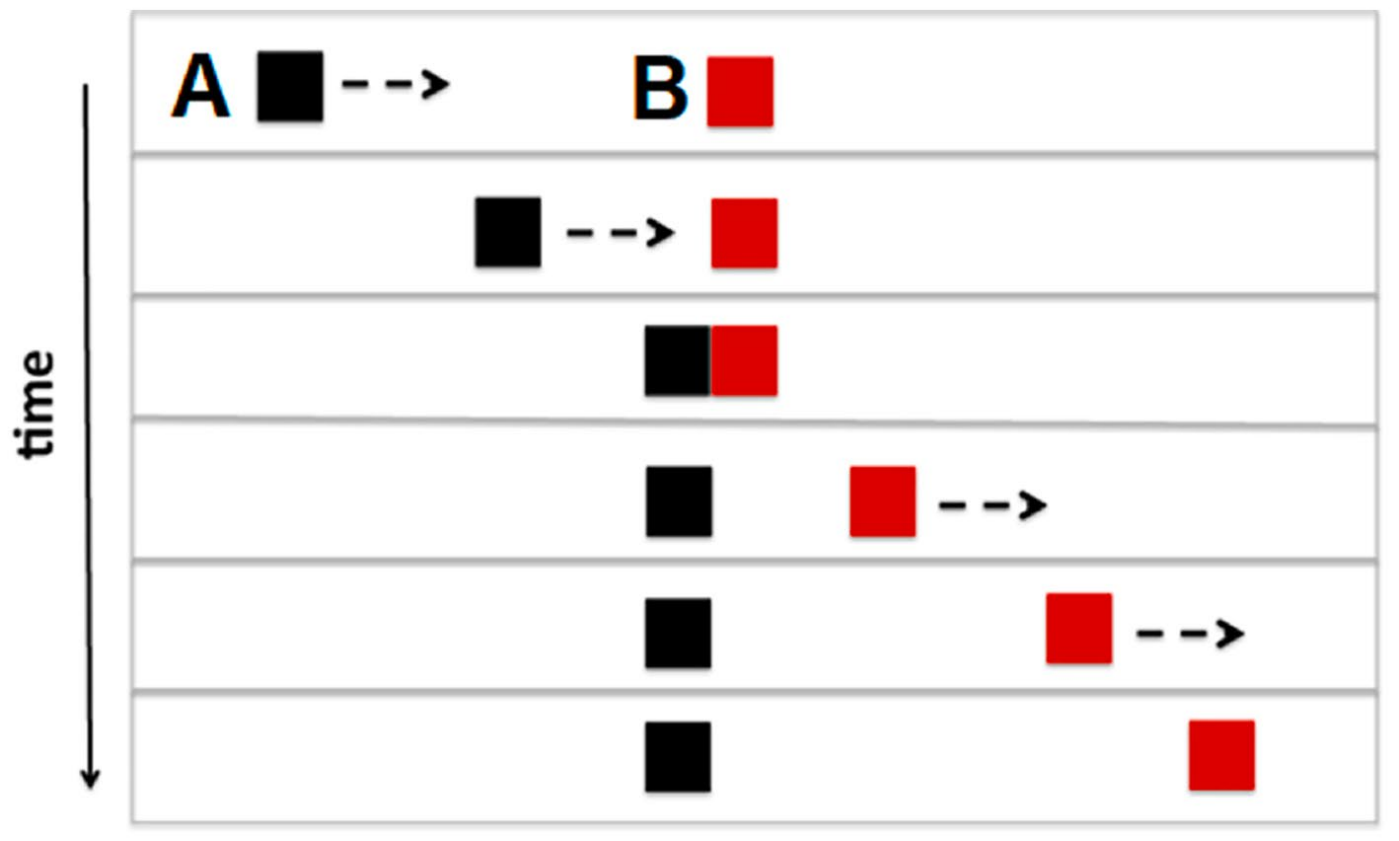
Six frames of *launching* or *triggering* animation, according to the speed ratio between square A and square B. Arrows indicate which object is moving in the various stages of the collision event.

In Michotte’s launching effect, when an object A moves toward and makes contact with another object B, B is perceived *as if it were pushed* by A in a mechanical collision (Michotte, 1946/1963; Thinès et al., 2014). According to Michotte, speed ratio and temporal contiguity are crucial factors in the perceptual organization of causality. If an interval is introduced between the two movements, it brings gradual changes in the launching impression; when the delay lasts too long (more than 140 ms), the causal impression disappears (Michotte, 1946/1963, p. 92; see Hubbard, 2013). With regard to the speed ratio, Michotte found that when the speed of the second movement exceeded the speed of the first movement (i.e., about twice as fast), the launching effect gives way to the *triggering effect*. In this case, the motion of the second square was sometimes seen as having an active and self-propelled character (demos are available at https://youtu.be/6r9meK27Tpw) (Figure 2).

Michotte (1946/1963) noted that the triggering effect was surprising – even comic. It was investigated further by Kanizsa and Vicario (1968), who found that when B’s motion is much faster than A, and B starts to move before the collision, B is described as if it were “seeing” the approaching object A and intentionally “escaping-away” from it. The authors named this action–reaction event as *intentional reaction* (see a demonstration here: https://youtu.be/BGjY61fzzd0). Observers spontaneously reported that the second object “runs away from the first,” such as “to avoid it,” or “because afraid” (Kanizsa and Vicario, 1968), thus exhibiting the presence of awareness and mental states even without goal-directedness (unless by goal we mean the intention to avoid a collision with the oncoming object). These and other similar impressions were later considered in infant and developmental research as a form of *psychological* or *social causality* (Schlottmann and Surian, 1999; Schlottmann et al., 2002, 2006, 2012, 2013; Schlottmann and Ray, 2010) or *action at-a-distance* (Spelke et al., 1995).

To investigate the relevant conditions for the perception of intentionality in adults, Dittrich and Lea (1994) designed experimental displays simulating a pattern of “one animal searching for another,” that is a pursuing relationship between moving letters in an array of distractors, and collected ratings about the presence of intentions, interactions, and animacy. They found that the impression of intentionality depends directly on variations in motion parameters, such as the direction and speed of the target’s motion, and the degree of goal orientation. Regarding the impression of animacy, they conclude that it may depend on both the presence of intentionality (i.e., goal-directedness) and the degree of interaction between the target and its goal.

A methodological limitation of these studies, concerns the choice of the dependent variables in the measurement of animacy and intentionality. While ratings or free descriptions of the visual stimuli are crucial to highlight the distinct phenomenology of these displays, they do not allow to separate the contribution of automatic visual processing from intervening higher-level reasoning processes based on such kinematic cues (see Firestone and Scholl, 2015; Van Buren and Scholl, 2018). For this reason, a relevant body of research focused on the psychophysics of *chasing* by adopting measures of visual performance that are better insulated from higher-level cognitive factors (Gao et al., 2009, 2010; Gao and Scholl, 2011; Gao et al., 2012) to better understand the interdependency between animacy and intentionality. It was shown that the human visual system is extremely sensitive in detecting chasing between two moving objects (a *wolf* and a *sheep*) in multiple objects displays. For example, Gao et al. (2009) introduced a specific methodological approach by employing visual search tasks in multiple object configurations and interactive displays. To control how directly the wolf approached the sheep, the authors varied the “chasing subtlety” – the maximal angular deviation of the *wolf*’s heading compared to perfect heat-seeking – and identified the optimal range of angular deviation from the straight chasing trajectory. Results indicated that with subtlety values above 30°, even when there was significant actual chasing, participants could not detect it. More generally, the perception of chasing is not a linear function of the degree of statistical correlation between wolf and sheep trajectories, but it depends on specific constraints.

In addition to investigations of chasing, Blythe et al. (1999) explored other basic categories of animate interaction, derived from evolutionary and ecological principles: pursuing, evading, fighting, courting and being courted, and playing. Interestingly, methodologically, the authors isolated the motion patterns of each category by means of a software built to allow subjects, interacting with each other across a computer network, to generate such behavioral trajectories. In this way, they extrapolated specific measures of trajectory parameters (velocity, vorticity, and energy) and plotted the most representative behavioral patterns.

From a theoretical point of view, while the energy conservation hypothesis implies that animacy can be triggered by simple movements that show an increase or change in their kinetic energy, other researchers suggested that the attribution of animacy involves – and may even require – something more than a failure in energy conservation, although this is necessary. Beyond motion, perception of animacy would be elicited by the inferences about the *causes of motion* – i.e., mental states, such as goal-directedness – in contrast to physical forces (Gelman et al., 1995; Premack and Premack, 1995; Opfer, 2002). In the perspective of these authors, even if triggered by particular combinations of visual features (Dittrich and Lea, 1994), the perception of intentionality and goal-directedness are strongly dependent on observer’s mental contents and inferential processes. According to Blythe et al. (1999), for example, the motion cues would activate simple heuristic and automatic algorithms necessary for the categorization of agents’ intentions. Many theories and computational models have been proposed to understand the development of causal cognition from infancy, based on the early predisposition to distinguish animate from inanimate objects from simple visual displays. In general, these approaches (e.g., Csibra, 2008; Baker et al., 2009; Gergely et al., 1995; Gergely and Csibra, 1997) strongly emphasize the role of rationality in understanding intentional behavior, such as the “teleological instance” or the “rationality principle.” They are not discussed here, however, as they are beyond the scope of this study.

For the sake of conceptual clarity, by the way, it may be helpful to emphasize that theoretical or methodological reasons have led many researchers to identify separate constructs that go beyond the main animate-inanimate or physical-social distinctions. These constructs are for instance goal-directedness, intentionality or agency. Nevertheless, one must be aware of the strong interdependence between basic perceptual constraints and higher-level social impressions. In fact, each of these cues may act independently to some extent, but in general they coexist and interact in the natural environment (see Gao and Scholl, 2011; Rosa-Salva et al., 2016). Chasing, for example, combines multiple motion and relational cues related to animacy, such as self-propulsion, acceleration, direction change, and target approach. Even from a phenomenological point of view, being a chaser cannot be separated from being alive, even if the relationship is asymmetrical. It has been suggested that the relationship between animacy attributions and mind attributions is not discrete, but may vary along a continuum from attributions of “physicality” – related to more mechanical characteristics – to attributions of “personhood” – related to human-like behavior (Santos et al., 2008).

Furthermore, as happens in the studies reported in the next section, attributing intentions and mental states to the moving objects would modulate and intensify the impression of animacy itself (Dittrich and Lea, 1994; Gelman et al., 1995; Premack and Premack, 1995; Tremoulet and Feldman, 2006): configurations with two or more moving objects appear more animated than those with a single object (Tremoulet and Feldman, 2006; Heberlein, 2008; Parovel et al., 2018).

For this reason, at least in research that investigates the complex relationship between abstract kinematic patterns and animacy, a number of authors have chosen to use “animacy” to refer to a general perceptual skill. Thus, the term animacy generally refers not only to basic lifelike impressions (i.e., self-propelled locomotion), but also to its related properties, i.e., the infinite nuances that go from animacy to intentional attributions. In most of the papers examined, the term animacy is combined with intentionality or animate agents for greater clarity, and they are often used as interchangeable terms (Gergely et al., 1995; Scholl and Tremoulet, 2000; Opfer, 2002; Tremoulet and Feldman, 2006; Heberlein, 2008; Santos et al., 2008; Visch and Tan, 2009; Gao and Scholl, 2011; Scholl and Gao, 2013; Rosa-Salva et al., 2016; van Buren et al., 2017; Kominsky et al., 2021; Lorenzi and Vallortigara, 2021).

## The influence of context: how the presence of a second object affects animacy judgments about the target object

To better explore the possible common ground between single movement patterns and social displays in the elicitation of animacy and intention, some works added other elements to the trajectory of a single object. Even in simple scenarios with a single moving object, in fact, another simple geometrical shape added to the display is enough to trigger an increase in animacy ratings. Tremoulet and Feldman’s (2006) displays showed a single figure (rectangular or round) moving on a screen and changing both speed and trajectory simultaneously while a static object (dot-foil or rectangular paddle) was placed in different positions. In this manner the static object defined several behavioral conditions for the target, such as moving *toward a prey* or *away from a predator* or being an obstacle. Tremoulet and Feldman found a small but significant effect of the context on animacy ratings, particularly in the goal/prey conditions. They suggested that a key factor in the perception of animacy is the attribution of an intention to the object - an intention that can be triggered by speed increase and change in direction alone, but that can also be specified by a supporting context.

More research was conducted to explore further the role of different spatiotemporal configurations on the perception of animacy and related properties, such as emotions and intentionality, in two-dimensional moving objects. In Parovel et al., 2018 work, the context consisted of a static or moving object that had the same shape as the target object (i.e., a small black square); in the dynamic conditions, the context object could exhibit either an animate-like (i.e., caterpillar locomotion) or a physical-like trajectory (bouncing event). The experiment was also designed to compare *approaching* vs. *avoiding* displays: it contrasted the relative directions between the target object and the context object. To obtain this, the context object could be located either at the beginning or at the end of the trajectory of the target (a sample of the stimuli is available here: https://youtu.be/4PyfhQoiVdk). Data were collected in both two-alternative forced-choice and Likert-scale rating tasks, and free reports were analyzed too. Results indicated a significant difference between static and dynamic contexts, where dynamic contexts prompted a distinctly clearer impression of animacy than static ones. Moreover, in the dynamic contexts it was consistently found that the impression of animacy was higher when the target was *moving away* from the context element than when it was approaching it. The moving-away behavior could be perceived as more animate for evolutionary reasons because of a higher sensitivity to threat-related events, such as fighting and chasing (Heberlein, 2008).

Psychophysical findings and free reports analysis suggested that there can be different facets to the animacy concept - for instance, an automatic animacy, an instinctive one and a mental/emotional one - and that an additional contextual element plays a crucial role in making them evident (Parovel et al., 2018). Visch and Tan (2009), in a similar perspective, showed that kinematic variations of abstract objects increase animacy attributions as well as specific emotional responses. For example, low velocity generating sadness and high velocity of the objects resulting in more “lively” movements. They speculated that animacy attribution is not only functional for social understanding and other adaptive purposes, but it also confers “reality status” and specific emotion correlations upon percepts of motion pictures. A recent paper confirmed the results of Heider and Simmel’s experiment with school-aged children and found that when the rectangular figure, i.e., the house, was present in the display, children produced a higher proportion of animated descriptions (Hofrichter et al., 2021). The overall results corroborated the theoretical assumption which states that intentionality and other emotions are “related properties” of animacy (Visch and Tan, 2009; Gao and Scholl, 2011; Scholl and Gao, 2013).

More generally, the attribution of animacy appears to be significantly influenced by the addition of other elements where the target object is moving: if a single moving object A begins to move, the only frame of reference available is the environmental one, for example the direction up or down or the speed change with respect to the background. If another - static or moving - object is placed next to the first, the same locomotion of A will be better specified by the type of their interaction. It can appear for instance as an intentional approaching behavior - prudent, or determined, according to its speed - or an avoidance behavior – again, more or less quick and compelling. Again, it seems uncertain whether a clear distinction between the animacy cues conveyed by the kinetic variations of single moving objects and those related to the interaction of multiple movements is functional for understanding the nature of the perceptual processes underlying animacy. In both cases, animacy impressions basically depend on a system of spatiotemporal relations: in the case of a single movement, the relationships connect the moving object with its immediate context - such as the environmental coordinates or a static object – while in the case of two or more moving objects, their interplay allows perceptual grouping in space and time.

## The role of spatiotemporal contingencies

In the vein of Michotte’s experimental work on perceptual causality (1946/1963), a significant amount of research has focused on the role played by the *spatiotemporal contingencies* between two or more moving objects in generating animacy (see Scholl and Tremoulet, 2000; Wagemans et al., 2006; Thinès et al., 2014). In examining the interdependence and similarity between animacy and causality, the temporal contiguity between the two moving objects is a common crucial variable, plausibly acting as a factor of perceptual grouping (Choi and Scholl, 2006; Duncker, 1935/1969; Schlottmann and Anderson, 1993; van Buren et al., 2017).

Bassili (1976) showed that temporal contingency was the crucial factor for the perception of an interaction between figures. Instead, spatial configuration of motion tended to determine the nature of that interaction, such as patterns of approach and avoidance. Because of the determinant role of temporal contingency, social interaction does not even require spatial contiguity. Schlottmann et al. (2002) and Falmier and Young (2008) found that *causality at a distance* – an action-and-reaction event similar to the intentional reaction- was easier to accept when the agents moved in an animate (caterpillar) manner and when the interaction was labeled as social (or psychological), rather than physical.

Another work, based on the manipulation of temporal contingencies between moving objects, showed that animacy experience increased with the time a moving object paused near a second object as well as with the increasing complexity of the interaction, such as approach and responsiveness, between the objects (Santos et al., 2008). Even a friendly/antagonistic communicative atmosphere can be induced by manipulating synchronous, coincident and not-coincident, movement of two egg shapes, on one side, and forward/backward/parallel tilting movement on the other (Yuasa, 2017).

In these situations, the movement of one object appears causally related to the movement of the other in a meaningful social relationship. In other cases, as it happens when two *casually* concomitant events are perceived *causally* related one to the other (see the example of the perceptual grouping between a door that shuts suddenly and the coming on of a light described by Duncker, 1935/1969), grouping can give rise to “incongruent” or “impossible” events, that may trigger even comic impressions. To assess if paradoxical causal contingencies between two trajectories that are incongruent and differently shaped are effective in evoking comicality, Parovel and Guidi (2015) combined Michotte’s launching configuration and locomotion cues. Precisely, they modified the pattern of the second phase of launching in different ways, to obtain animated trajectories, such as a *frog-like* expanding and contracting trajectory or a *rabbit-like* jumping trajectory, as well as physical trajectories, such as *rotating* and *bouncing* squares (a sample of the stimuli is visible at: https://youtu.be/5EeihxEHdiY). The authors found that the paradoxical juxtaposition of animacy cues inside a launching relationship – while not of incongruent physical trajectories – elicited in the participants comical appreciations, in line with the Bergsonian theory of humor (for similar results see also Bressanelli and Parovel, 2012).

Results from Parovel and Guidi (2015) showed that temporal contingency has a crucial influence in evoking comic impressions: scale values and ratings of comicality actually tended to decrease with an increasing delay between the two movements. With a 200 ms temporal delay, it was still possible to get an impression of paradoxical causality between the two movements whilst, with a 1 s delay, perceived causality was disrupted. Interestingly, when spatiotemporal conditions convey an impression of psychological causality (−200 ms delay, and the speed of the first movement lower than the speed of the second movement), even linear trajectory events are judged amusing, confirming the previously quoted Michotte’s observation about triggering (Michotte, 1946/1963).

Also, in other combinations of interacting moving objects, it has been observed that a change in the kinetic behavior of one object – i.e., a pause – elicits the perception of animacy only when a second object intercepts its trajectory in coincidence with the pause: the discontinuity in movement is then perceived as an intentional “waiting” (Minguzzi, 1961). Reasonably, kinetic conditions alone are not unequivocal and specific and so easily influenced by some other properties (Gyulai, 2000).

## Interim summary

The reviewed research has revealed the existence of multiple visual parameters inducing the observer to attribute animacy in moving objects. The term animacy, as we have seen, generally refers not to one specific impression, but also to a whole range of nuances of meanings. Consistently, with regard to the complexity of the scenario, these meanings can run from autonomous activity to emotional states such as, for example, fear or curiosity, as well as to psychological intentions, such as aggressive or shy, avoiding or approaching.

Moreover, a large body of psychophysical evidence, since Michotte’s and Heider and Simmel’s animations, has demonstrated a close dependence of animacy impressions on the spatiotemporal conditions of the stimulation. Therefore, as already seen, minimal variations of physical parameters correspond to discrete differences in the impression of animacy (Santos et al., 2008).

What clearly emerges is that, whilst observing such events, it is almost impossible to perceive the movement as neutral and meaningless, suggesting that the visual system is directly tuned not so much to the “objective” stimulation, but rather to the meaningful information conveyed by these movements – information of high adaptive and behavioral value. Detecting the presence and understanding the intentions of other agents is crucial to survival and reproduction. Thus, it is plausible that humans and other species evolved to be extremely sensitive to signals of animacy and agency, and that they possess fast and unlearnt mechanisms for the detection of them (Mascalzoni et al., 2010; Vallortigara, 2012; Abdai et al., 2017; Lorenzi and Vallortigara, 2021).

Although this paper does not incorporate neuroscientific evidence, it is important to mention that the neural substrates associated with animate motion processing are at least in part distinct from those associated with inanimate motion. The exposure to such visual stimuli elicits strong activation in the temporoparietal cortex, including areas in and near the posterior superior temporal sulcus (pSTS) and angular gyrus, especially in the right hemisphere (Castelli et al., 2000; Blakemore et al., 2003; Heberlein, 2008; Santos et al., 2010). Some data showed that the ability to detect animacy from contingency of objects reacting to other objects is processed by specific networks which are different from brain regions associated to theory of mind tasks. The detection of agency on the basis of cues such as movement and contingency, according to these authors, might be a precursor of our ability to infer other people’s mental states (Blakemore et al., 2003). Additionally, to explain the neural substrate underlying the understanding of animacy, two hypotheses have been proposed: the mirror-system hypothesis (Gallese et al., 2004) and the social-network hypothesis (Adolphs, 2003; Wheatley et al., 2007), each engaging anatomically distinct neural substrates (see for a review, for instance, Slotnick, 2013).

## Is animacy directly perceived?

Is animacy a visual property, like shape and size, or the result of automatic reasoning? The “place” of animacy and causality in our perceptual experience is a central theoretical question in experimental psychology and cognitive science. It concerns the complex relationship between perception and cognition.

The nod of debate can be formulated in these terms: (a) do we see low-level cues evoking top-down perceptual judgments about animacy or, (b) interactions themselves are meaningful, as stated by Michotte, because they are the result of bottom-up features within visual processing?

A major obstacle to finding a convergent solution is posed by the fact that the existing positions in cognitive psychology are based on different theoretical premises concerning the nature of the information available to our senses, the so-called “thin” and “rich” views (see Toribio, 2018). According to the “thin” view, the texture - or information - available to the sensory system is limited and insufficient to specify the properties and the events of the world. Thus, animacy impressions - even when elicited by low-level features such as color, shape, texture, or motion - would depend on high-level inferences drawn from information present in long-term memory (see for reviews Rips, 2011; Scholl and Gao, 2013). This is not to say that these properties do not have a compelling appearance as objective properties, but that their phenomenology is insufficient to prove their true low-level nature. Spatial, temporal, and other visual cues would be processed and automatically detected, and this immediacy would be erroneously attributed to visual processing itself. While watching Michotte’s like demos, observers would activate representations – or schemas – and expectations in long-term memory relative to the ongoing events in order to recognize them (see Weir, 1978; White and Milne, 1997; Tenenbaum and Griffiths, 2003; White, 2006). These schemas are post-perceptual and, according to these theories, can be acquired with experience and modified by beliefs and expectations.

Otherwise, the so-called “rich” view upholds the hypothesis that the visual system can directly detect meaningful relationships and interactions between objects. This view has its foundations in Michotte’s claim, and it was later reformulated as a thesis about a module-based perception of animacy and causality in line with Fodor’s perspective, and received much empirical support from, among others, Scholl’s research group (see for reviews Scholl and Tremoulet, 2000; Scholl and Gao, 2013). Such perceptual modules would be informationally encapsulated and therefore not shaped by prior knowledge, inference, or expectation (Saxe and Carey, 2006). According to “rich” theories, high level properties are visually represented and not just seemingly represented as a result of a perceptual judgment (Rips, 2011; Toribio, 2018). This position is compatible with the idea that observers have specialized detectors, hardwired in the perceptual system, to take over physical, biological and social interactions (Vallortigara, 2012). Perceptual animacy and causality may occur on first exposure without requiring prior experience with the events. Further learning would take advantage of this elementary, original knowledge and would shape more sophisticated cognitive skills and behaviors (Lorenzi and Vallortigara, 2021).

The main arguments that have been put forward for and against the different positions, i.e., the “perceptual” view and the more widespread “top-down” view, will be briefly mentioned in the following paragraphs. Then, we will address three additional findings in support of the involvement of automatic visual processing in the impressions of animacy: the evidence in favor of a sensitivity to animacy in newborns; the lifelong persistence of animacy through motion despite visual incongruity; the interactions between animacy and other visual processes that have been recently documented.

Scholl and Tremoulet (2000, p. 299) claimed that causal relations and animacy are rich and vivid properties of visual displays, and are “fairly fast, automatic, irresistible, and highly stimulus-driven” phenomena. The phenomenal character of *vividness*, however, is not a valid argument, since it is recognized by both approaches. The same can be said for the apparent effortless and unawareness typically associated with the perception of animacy and causality. Many post-perceptual judgments can also occur effortlessly, automatically, and unintentionally, and even other cognitive processes unrelated to vision (e.g., semantic priming) share these properties without being hardwired in the early stages of visual processing.

On the other hand*, fastness* and *automaticity* can be plausibly related to the adaptive role of this sensitivity, i.e., the satisfaction of vital biological and social needs. Vallortigara (2012) conjectured the existence of a sort of perceptual “life detector” in the brain, inspired by Darwin’s suggestion about primitive neural pathways to ensure a bias to attend toward living things. Behavioral and neuroscientific evidence for an innate predisposition to animacy cues comes from research on animal and human newborn cognition. Such data supports the idea that the selective pressure to quickly detect and respond to the presence of other creatures has shaped the brains and behaviors of distant animal species in similar ways throughout evolution (see, for a recent review, Lorenzi and Vallortigara, 2021). It is highly advantageous for animals, the authors argue, to be born with preprogrammed mechanisms for directing attention to salient categories of stimuli, such as animacy and agency, rather than having to learn them through long sequences of trying and failing. This also could lay the groundwork for further refinement as development proceeds.

Furthermore, most of the findings in this area show “dramatic effects of very subtle stimulus manipulations. This is a hallmark of perception” (Scholl and Gao, 2013, p. 198). In other words, the very close *dependence of causality and animacy impressions on the objective spatio-temporal conditions* of the stimulation – such as distance, duration, speed of movement, and so on -, would have been extremely difficult to explain on the basis of previous knowledge and experience (Runeson and Frykholm, 1983; Santos et al., 2010). Similarly, according to Butterfill (2009), observer’s sensitivity to some causal interactions and dependency on very brief temporal delays is properly perceptual and categorical, just as phoneme perception is. According to Butterfill, however, the role of causal categorization would not be to convey information about the nature of the event, but only to allow the observer to distinguish different events from each other.

Another classic argument, since the work of Michotte (1946/1963) and Heider and Simmel (1944), concerns the *unanimity of subjective reports* and their inconsistency with individual differences.

Some studies found individual differences in their investigation on causal impressions (Gemelli and Cappellini, 1958; Beasley, 1968; Young and Falmier, 2008). However, many of these studies are difficult to evaluate because they do not clearly separate perceptual processing from other effects, such as those due to task design or other uncontrolled individual dispositions (Rips, 2011). In order to overcome this problem, some authors have preferred to avoid methods like ratings and individual judgments – that appear to be particularly susceptible to being contaminated by post-perceptual judgment (see Firestone and Scholl, 2015; Van Buren and Scholl, 2018), adopting tasks that measure visual-motor performance (Blythe et al., 2009; Gao et al., 2009) or two-alternative forced-choice comparisons (Parovel et al., 2018).

The *irresistibility of the stimulus*, i.e., the cognitive impenetrability of these impressions, is a further criterion that has been proposed to support the hypothesis that animacy is genuinely perceived and not the result of a perceptual judgment (Scholl and Tremoulet, 2000). The impression of animacy is compelling and irresistible; thus, animacy and related properties can be assimilated to standard visual illusions, whose appearance persists even when we know about their objective conditions (see Pylyshyn, 1999). Toribio, in her theoretical work (2015), argues that the irresistibility of the stimulus criterion is the most important evidence supporting the visibility thesis for high-level properties like animacy. Specifically, she refers to the experimental results of Gao and Scholl (2011), which imply the use of information that is not available to the subjects, thus ruling out any effect of top-down inference. More generally, Toribio notes, subjects are well aware that the geometric shapes in motion on the screen are not animate. However, even in the face of conflicting background knowledge, under certain conditions they cannot help but experience such characteristics.

More skeptical is the position of Rips (2011), who provides a theoretical overview of all the work on causal processing along the whole range of conditions, from mechanical launch to animacy and intentionality attributions. Using data from infant and animal studies, cognitive and neuropsychological dissociation experiments, and studies of context effects and individual differences, the author contrasts the two main explanatory hypotheses: the cause-detector hypothesis – a reformulation of the perceptual module hypothesis – and the causal schema hypothesis (Weir, 1978). In the latter, representations or schemas of simple interaction patterns (e.g., launching, triggering, pulling, and so on) are the result of non-modular inferences based on long-term memory information.

The two models, Rips (2011) argues, differ on many assumptions, such as information encapsulation, innateness, the role of development, individual experience and cultural differences, but not on others, such as fastness, automaticity and unanimity. For this reason, much of the evidence does not allow for a distinction to be made between the two competing theories. In any case, in Rips’s view, module evidence does not mean that we perceive causal events, but rather spatiotemporal relations that are informed by higher-level knowledge.

A recent study by Morales-Bader et al. (2020) emphasized the role of semantic cues and high-level processes in animacy judgments. They suggested that the tendency to attribute intentionality in Heider and Simmel-like displays can be affected by the interaction between perceptual and semantic cues (i.e., figure shape, label, and apparent speed). Interestingly, by the way, when the authors contrasted the effect of the figure’s shape on the attribution of intentionality, they found that triangular shapes were attributed more intentionality than anthropomorphic-stickman figures. This was interpreted as anthropomorphic figures acting as distractors to the type of movement, which was the main cue that led to animacy and intention attributions. Unfortunately, as mentioned above, the methodology adopted by the authors, i.e., categorizing participants’ free descriptions, does not allow separating the role of genuine visual processing from the intervention of automatic inferential judgments.

Furthermore, the empirical evidence provided by developmental studies, shows that perceptual causality may be available early, at a time when relevant experience is limited, while the simplest form of causal reasoning develops much later (Schlottmann et al., 2002; Saxe and Carey, 2006). The view of Schlottmann et al. (2006) to explain this gap is that perceptual causality is useful early in children’s development because it allows identification of causal events for themselves without need to reason about “why” these events are cause and effect. In fact, children cannot always integrate perceptual constraints with causal mechanisms – the underlying structure of events – until later in development. Generally, according to these authors, developmental evidence cannot exclude a post-perceptual role for higher-level knowledge and learning (see also Vicovaro, 2018).

The presence of animacy-related kinematic constraints from the earliest days of life and their developmental function, in contrast to the appearance-based visual features associated with animate beings, is another developmental issue worth exploring in more detail. It will be partially covered in the next section.

## What newborn babies are attracted to: animated movement and face-like patterns

Ample empirical evidence from infant research supports the notion that from birth the human system is broadly tuned to detect social stimuli on the basis of at least two independent properties: the presence of a face and the way something is moving. Nevertheless, the ontogenetic origin of this sensitivity is still under debate.

Evidence from fMRI literature on functional characterization of cortical responses in infants demonstrates that the cortex of 4–6-month-old human infants is already spatially organized, with distinct regions responding preferentially to human faces versus natural scenes (Deen et al., 2017).

Many data have shown the existence of mechanisms that ensure that newborns’ attention is triggered by faces, and that they manifest preference for schematic and real faces. With regard to face-like patterns, it has been shown that newborns look more frequently and longer at geometric stimuli with more elements in the upper part when compared to the inverted version. This preference, would allow newborns to successfully choose faces from other non-face-like stimuli (Simion et al., 2002; Turati et al., 2002; Macchi Cassia et al., 2004).

It has also been recognized that infants are predisposed to attend preferentially to the motion of biological entities, even when presented in the most rudimentary form. These predispositions are thought to be controlled by rapid and automatic subcortical orienting mechanisms, and their presence at birth would contribute to the development through progressive specialization - as a function of experience - of the “social brain” network (Troje and Westhoff, 2006; Yoon and Johnson, 2009; Simion et al., 2011; Di Giorgio et al., 2016).

The first strong evidence for an innate ability to detect biological motion and to respond to it in a specific way came from non-human animal species. Imprinting procedure revealed that newly hatched chicks at their first exposure to point-light displays preferentially approached biological motion compared to nonbiological motion stimuli (Regolin et al., 2000). Moreover, the data suggest a non-species-specific sensitivity to biological motion, as chicks showed no preference for the patterns displaying a walking hen over configurations displaying a walking cat or even scrambled biological motion, all of which are preferred over stimuli that do not display animated motion (Vallortigara et al., 2005; Vallortigara and Regolin, 2006). Similar to adult human observers, visually naive chicks also showed a significant preference for moving stimuli that changed speed relative to stimuli with constant speed, and for stimuli that changed direction (Rosa Salva et al., 2015; Rosa-Salva et al., 2016). Recent neuroscientific evidence has revealed the involvement of subcortical areas of the avian brain in response to stimuli showing speed changes, as compared to those showing constant motion (Lorenzi et al., 2017).

In humans, it has been shown that 4- and 6-month-old infants respond to biological motion stimuli, as they tended to look longer at a point-light display of a walking person than at an array of randomly moving elements (Fox and McDaniel, 1982).

More recent results demonstrated sensitivity for the dynamics of biological motion and to the gravitational forces acting on motion even in newborns. To rule out the possibility of any previous experience, authors adopted hen-walking animations rather than human-walker animations and found that at their first exposure, 2-day-old babies preferred biological motion over random motion point-light displays (Simion et al., 2008; Bardi et al., 2011). Furthermore, newborns choose the upright point-light display of a walking hen over the same display inverted.

Changes in speed seem to be relevant as well, as recent developmental studies demonstrated. When presented with speed changes, newborns showed a preference for a particular speed pattern, i.e., an increase followed by a decrease in speed. In contrast, the reverse sequence pattern or a single speed change do not elicit any visual preference (Di Giorgio et al., 2021).

Regarding the perception of visual features other than the face in the first weeks of life, findings on infant visual categorization development – obtained by comparing behavioral data (i.e., the looking behavior) and brain-activity recording-, suggest that animacy is the earliest categorical distinction of visual objects in infancy (Spriet et al., 2022). However, until 4 months of age, infants’ looking behavior when presented with a series of images of real-world objects, shows no evidence of an animate-animate distinction, while revealing a preference for size, elongation, and compactness of objects. Four-month-old infants continue to prefer human and nonhuman faces and bigger objects, but they also show categorizing by animacy. By 10 months, image categorization by animacy emerges despite differences in image size and it is consistent with the cortical organization of object-related information recorded from anterior (temporal) aspects of the visual ventral stream in adults (Spriet et al., 2022).

In summary, it has been theorized that the ability to perceive a wide range of face types, regardless of species specificity, is of great adaptive value for infants (Nelson, 2001). In a similar way, it could be hypothesized that an inborn broad sensitivity to life-like movements in infants – a life detector – could be very advantageous in directing attention toward living things and in differentiating them from inanimate (Vallortigara, 2012). It would allow infants to discriminate between differently shaped entities and patterns, providing crucial support for visual experience in the development of categorical inferences, animacy-related, as well as responses to primitive perceptual features – such as mid-level features. It has been suggested, in fact, that much of object-selective cortical organization can be explained by relatively primitive mid-level features without requiring explicit recognition of the objects themselves (Long et al., 2018).

More generally, a broad sensitivity to animated motion would have the advantage of great flexibility and attention to an equally broad range of possible events. The possibility of committing biases in the sense of attributing life to non-animated objects would be well compensated by avoiding the opposite – and worse – risk.

## Falling leaves or butterflies? An aesthetical side effect of the *irresistibility* criterion

It remains yet to be clarified how these innate mechanisms evolve with the development of more complex and detailed cognitive capacities. According to several authors, perceptual narrowing would occur for instance with increasing experience with certain types of faces and lack of exposure to other types of faces. This would allow the human system to increase its ability to discriminate the highly experienced faces and decrease its ability to discriminate the infrequently experienced faces (Nelson, 2001; Simion et al., 2011). Also, considering perceptual causality, it has been argued that it is an innate tool with the role of supporting learning about the causal texture of the world, and then learning gradually influences perception (Schlottmann et al., 2006).

Nevertheless, much of the literature reviewed in the first sections of this work emphasizes the persistence and automaticity of these impressions into adulthood, despite the knowledge acquired through learning (i.e., the *irrestibility* criterion). If the acquisition of more complex cognitive skills leads to the merging of perceptual constraints and learned mechanisms, then the acquired knowledge about physical mechanisms and social behavior of organisms should prevent adults from juxtaposing mechanical and animate features in perceptual events. On the contrary, animacy impressions take place even in conjunction with relatively incongruent visual information such as geometric moving shapes, remain vivid after many repeated observations, are easily induced and surprise - and fascinate too - the observer. This is well represented by many side effects, other than animated cartoons with non-anthropomorphic forms: we all experience the erroneous attribution of life to inanimate moving objects in the natural environment, e.g., when a leaf blown by the wind seems a living creature - like a butterfly. Or, when a very elastic bouncing ball seems to jump. Similarly, familiar non-living objects can appear alive in movies created by “stop motion” techniques (i.e., a filmmaking technique in which an object is moved in small steps and a photograph is taken at each step), or by performing other video editing operations, such as rewinding playback – which can, for example, make a dropping object appear to rise up against the force of gravity. The pleasure elicited by puppets and marionettes may be another side-effect of the persistence of animacy through motion despite visual incongruity. Actually, many great thinkers have emphasized the importance of juxtaposing, in the same event, mechanical and animated visual qualities to achieve a comic or surreal effect (Bergson, 1900/2013; Ejzenstejn, 2004).

A similar side effect can be speculated to occur in the perception of faces, as suggested by the common phenomenon of pareidolia, the tendency to perceive a face even in a non-living object, such as the moon, clouds, rocks, or the front of a car, a phenomenon described since Leonardo Da Vinci (see for instance Ichikawa et al., 2011). Even in these cases, we see both the objective and inanimate nature of the object and the manifest presence of the face at the same time. Not only that, but we cannot help but even see that the face expresses psychological traits.

This evidence suggests the lifelong persistence of these innate mechanisms and their independence, at least in part, from developmentally acquired inference and categorization processes, thus allowing flexible adaption to changing circumstances (Schlottmann, 2001). In presence of purely casually contingent (or intentionally induced) animacy-related kinematics, learning and reasoning warn us and prevent us from inferring “objective” causality or animacy in the visual scene. On the other hand, reasoning, fortunately, does not prevent the aesthetic enjoyment of surprising and of vivid paradoxical effects when they occur, by choice or by chance. Given the crucial importance for human - and not only human - observers of the detection of life and agency, it is plausible to speculate, as some authors have argued, that we have evolved to be very sensitive, or even *overly sensitive*, to animacy and agency (Vallortigara, 2012).

## Interactions of animacy with other visual processes

As seen in the previous sections, animacy and intentional relationships between moving objects are extracted rapidly and automatically, are sensitive to subtle visual parameters, appear early in development and are present in non-human species. For these reasons, these phenomena would show important hallmarks of automatic visual processing (see Scholl and Gao, 2013). Recently, some authors have further challenged the top-down perspective and claimed that, by virtue of their ecological and adaptive relevance, the perception of animacy and intentionality may be integrated into the mind in ways that are deeper than previously imagined. They hypothesized that animacy may interact with other perceptual processes (van Buren and Scholl, 2017; Hafri and Firestone, 2021). If perceptual animacy can influence other low-level visual features, this should be a further evidence supporting the theory that animacy is processed in the earliest stages of vision and is not a high-level projection added by the observer’s mind to neutral stimuli.

In the last two decades abundant empirical evidence has emerged supporting that the perception of launching events can have an influence on other processes in visual cognition. The following paragraphs will first summarize these works and then mention some recent findings on new specific interactions between animacy and other visual processes.

It has been shown that the launching effect can imply: (a) a contraction (two objects appear closer in space when they are causally connected; Buehner and Humphreys, 2010), or (b) an extension of the perceived distance between the colliding squares A and B at the moment of impact, i.e., the degree of overlapping between the two items is underestimated and the degree of underestimation is higher when the causal nature of the event is induced by a surrounding context (Scholl and Nakayama, 2004); (c) a distortion of the perceived trajectory of the apparent motion of A (Kim et al., 2013); (d) larger displacements in the remembered vanishing position for moving targets when the launcher was faster than the launched object (Hubbard et al., 2001; Hubbard and Ruppel, 2002; Choi and Scholl, 2006; De Sá Teixeira et al., 2008); (e) a distortion of the remembered temporal order of the motions of the squares A and B (Bechlivanidis and Lagnado, 2016). Apparent kinematics itself, (f) can be biased in launching events: in certain conditions the perceived speed of B is influenced by the speed of A (Parovel and Casco, 2006; Vicovaro et al., 2020). Causal relations are also visually “prioritized “in the following ways: (g) participants become aware of causal events more rapidly than non-causal events (Moors et al., 2017), (h) launching events are subject to retinotopically specific visual adaptation (Rolfs et al., 2013), and (i) in visual search tasks, adults’ causal perception distinguishes between triggering and launching events and this ability cannot be attributed to low-level differences in sensitivity to differences in speed. Instead, according to these authors, this categorical boundary is directly determined by constraints on perception (Kominsky et al., 2017).

Concerning the influence of perceptual animacy on other visual processes, the following paragraphs will discuss, respectively, visuomotor performance, visual memory and speed estimation.

A series of studies has demonstrated that animacy cues influence visuomotor performance (Gao et al., 2010). For example, in various interactive tasks in which participants controlled a disk within a display filled with randomly moving darts, the task of “avoiding” from a pursuing “wolf” disk was less successful if the randomly moving darts remained oriented straight toward the subject’s disk (*wolfpack* displays). According to the authors, this happened because in this condition the wolfpack darts were perceived (erroneously) as actively pursuing the subject’s disk. Van Buren et al. (2016) explored whether such displays would influence performance even when the putatively animate objects were entirely irrelevant to the task, and subjects were asked to ignore them. Also in this case, subjects took longer to perform their task – to collect dots as quickly as possible – when the irrelevant background darts were always pointing at the disk they were controlling, rather than 90° away from it.

Following the same line of investigation, Van Buren and Scholl (2017) explored the influence of perceived animacy and goal-directedness from simple geometric shapes on spatial memory performance. In particular, they wondered if a matching task between pairs of wolfpack panels, in which participants saw animations with both “darts” and discs with sketched “eyes,” would be influenced by animacy cues. Results showed a spatial memory advantage for stimuli that were perceived in animate and intentional terms, and these effects occurred both with “darts” and “eyes.” The authors emphasized that the wolfpack panels were prioritized in memory over all other types of panels, showing a robust effect; they suggested that perceiving animacy can really matter for downstream processing.

Finally, a recent work discovered an illusory speed effect in displays conveying animacy (Parovel and Guidi, 2020). A first experiment was based on previous research reported above (Parovel et al., 2018), which found that (a) a moving square created an impression of greater animacy in dynamic contexts than in static ones, and (b) when the target moved away from the context element than when it approached it. In this work, instead, two-alternative forced-choice comparisons were used to test whether the perceived speed of the target square varied across the same set of stimulus conditions. Results showed that an escaping object looked *faster* than an approaching one or neutral one, moving in absence of any context (some demos are available at: https://youtu.be/p17c41B_lq8). In a second experiment, the perceived speed of the escaping black square was psychophysically measured in a condition similar to the *intentional reaction* (Kanizsa and Vicario, 1968), where a two-dimensional square moves toward another square, which gets away before the first square reaches it. The point of subjective equality (PSE) estimates indicated that the speed of the escaping moving object was overestimated between 6.7 and 10.2%, according to the type of motion of the chaser (*linear* vs. *caterpillar-like*). In conclusion, the speed overestimation was found only in the escaping condition and not in the approaching one, and it was stronger when the contextual element, the chaser, moved like a caterpillar.

To summarize, the empirical evidence described in this section - analyzing the influence of animacy on visual performance, visual memory, and speed perception – provides further support to the hypothesis that animacy perception is hardwired in the visual system (see Hafri and Firestone, 2021). It is interesting to note how all the interactions reported above between animacy and other visual processes are concerning “chasing” situations, i.e., potentially threatening events that would therefore require an immediate behavioral reaction. If there exists a perceptual “life detector” hardwired in the brain (Vallortigara, 2012) overall, it seems extremely plausible that it should interact rapidly and efficiently with other visual abilities, favoring appropriate visual-motor skills to quickly react with adaptive behaviors to the surrounding events.

In addition, social relationships involving interacting human figures exhibit further perceptual specificities (see Hafri and Firestone, 2021). To mention just a few findings in this area: (a) extraction of event structure from visual scenes is rapid and spontaneous, as shown in dynamic sequences of two-person scenes, designed to distinguish actors from patients (Hafri et al., 2018); (b) visual search advantage found for face-to-face, relative to back-to-back dyads (Vestner et al., 2020); (c) interacting individuals are remembered as physically closer than are noninteracting individuals (Vestner et al., 2019); (d) meaningful interaction between human agents helps working memory to compress the movements to be stored into a chunk (Ding et al., 2017); (e) visual adaptation aftereffects have been reported suggesting selective coding mechanism for action contingencies (Fedorov et al., 2018).

More generally, all these data are consistent with similar findings on the attentional visual prioritization found in detecting animate objects, using natural looking images (Altman et al., 2016; Bailey and Lang, 2022; Long et al., 2018). The discoveries made in this field are generally interpreted as an additional support for the animate-monitoring hypothesis (New et al., 2007), which suggests that early detection of animacy may have endowed our hunter-gather ancestors with survival advantages, by means of perceptual features that have remained consistent throughout hominid evolution.

## The life-detector’s role: a broad-range sensitivity to the ongoing changes in a multiple relational system?

As seen so far, several lines of research support the hypothesis that animacy and its related properties are hardwired in the brain and are automatically processed in the earliest stages of vision. According to these findings, animacy features – originally defined as kinetic structures by Michotte (1946/1963), or spatiotemporal gestalten by Heider and Simmel (1944), likely lend themselves to being conceived as prelinguistic visual primitives (Mandler, 1992) or as social affordances, whose meaning can be directly perceived and solicit the animal’s behavior and affect (Gibson, 1977; Withagen et al., 2012; Withagen, 2022), rather than as the top-down result of higher-level processes of recognition and categorization.

In this perspective, what is still unknown is if the numerous kinds of animacy and social interactions are modular specific or depend on a unitary animacy-detector system. In the first case, infants would exhibit separate core systems for animate and inanimate objects (Leslie, 1994; Premack, 1990; Mandler, 2003; Spelke and Kinzler, 2007; Vallortigara, 2012; Scholl and Gao, 2013). In the second case, some theoretical frameworks, such as the cue-based-bootstrapping model, speculate that innate predispositions to low-level visual cues linked to animate beings lead to the development of animacy perception through learning (Schlottmann et al., 2006; Biro et al., 2007; Spriet et al., 2022). Since birth, humans would display some attentional biases toward rudimentary low-level visual cues of motion – such as start from rest by self-propulsion and speed changes - that elicit animacy perception also in adults (Di Giorgio et al., 2021). The exact nature of such low-level cues, i.e., whether the information they carry can be considered only an initial precursor, or whether it is inherently significant information about animacy, is still uncertain.

A further open question concerns whether the cues eliciting the impression of animacy belong to specific categories, such as animate-inanimate (Kominsky et al., 2017). One possibility, proposed by Gao and Scholl (2011), is that different kinematic cues would correspond to specific animacy categories, such as triggering, chasing, approaching, and so on, and all these individual cues could be observed – and investigated – either in isolation or mixed together in common displays. Anyway, although psychophysical experiments usually isolate animacy-inducing parameters, their interaction – for example in more complex displays or in real life situations - can produce results that are not additive and therefore not predictable (Tremoulet and Feldman, 2006). In general, the existence of a specific module for perceiving each form of animacy seems problematic to maintain (see Rips, 2011). Even if perceptual relations generally reflect distinct categories (see Hafri and Firestone, 2021), both phenomenology and experimental psychophysics suggest that the animacy construct must be defined in a broad sense. Given the theoretically infinite range of animacy-related impressions, animated and social events may be better understood not as all-or-nothing properties, but by allowing for the possibility of intermediate categories that are perceptually meaningful.

In this view, the ability to grasp animacy in all its nuances could be understood as a broad-range sensitivity toward those characteristics of kinematics which involve the presence of living beings and agents. This would allow for not only recognition of their presence and of their animate movements, but also to quickly grasp their psychological, emotional and interpersonal characteristics (for instance, being calm, hasty, friendly, avoiding, nervous, unsure, edgy, etc.). Perhaps even moral instances such as helping or hindering, i.e., altruistic versus selfish behavior, can be directly captured and differentiated, as Kuhlmeier et al. (2003) have shown in 12-month-old infants.

This wide-range sensitivity could be rooted in the predisposition of the visual system to perceive spatiotemporal relationships between movements that are intrinsically endowed with information about the nature of the ongoing events. Indeed, across physical and social domains, current findings and theories have reinforced the possibility that meaningful relationships between movements are properly perceived for themselves and reflect highly specialized visual processes (Van Buren et al., 2016; Hafri and Firestone, 2021).

In summary, what characteristics must a moving object, or a global kinematic pattern, have in order to trigger the animacy response of a hypothetical life-detector broadly tuned to meaningful relationship?

As Aristotle wrote, if there is no external force putting it into motion, a moving object appears as having an inner force, i.e., life. Considering all the nuances of animacy that may appear in different types of interaction, we can speculate that besides this biological force, other apparent causes, namely psychological and social, may emerge from kinematic displays. From this perspective, multiple cues must be considered in order to search for a common perceptual sensitivity that might encompass and integrate them together.

As previously seen, a life-detector should be sensitive (a) to the onset/presence, to the changes in speed of already self-propelled moving objects (Lorenzi and Vallortigara, 2021). Furthermore, in animal and human locomotion, (b) a life-detector has to be able to identify the relationships between the constitutive parts of the object, such as the head and tail – e.g., in caterpillar-like non-rigid stretch-and-squeeze motion–, as well as the interaction between several individual points – e.g., in a biological movement pattern. Indeed, in the movement of vertebrates, the spatial relationships between some parts of the body are constantly changing, while the spatial relationships between other parts, which represent connected joints, remain invariant. In other words, the moving object has to be related to structural invariants, such as semi-rigidity principles versus the spatial constancy displayed by rigid inanimate objects (Simion et al., 2011).

Moreover, a moving object, in order to trigger the animacy response, (c) has to be related to physical constraints, such as force of gravity and energy conservation principle (Jörges and López-Moliner, 2017). In addition, (d) a life-detector must keep into account the visible changing relationship between a moving object and its environmental coordinates (movement direction, shape of the trajectory); it must be able (e) to detect the interaction between one moving object and other elements (e.g., avoiding an obstacle); it has to recognize (f) the interaction between two or more moving objects (chasing, approaching, or other social relationship).

Spatiotemporal contingencies (g) are another crucial cue in modulating social and psychological meaningful patterns, and in distinguishing causal from casual interactions. In other words, movements between agents have to look functionally related, that is, the changes of the one must appear as directly dependent on the changes of the other, at least within a specific range of variations (Michotte, 1946/1963; Gao et al., 2009). Very interesting in this regard is the recent work of Lemarie and colleagues (2022), who have emphasized the significant role of a certain degree of unpredictability in the temporal coincidences between interacting moving stimuli in domestic chicks. Animate agents, the authors argue, might require imperfect spatiotemporal contingencies between interactive moving objects – differently to launching events – and might avoid the perception of ‘repetitive’ or ‘mechanical’ movements in social aggregation stimuli. Similarly, the irregularity and unpredictability of individual trajectories can be understood as lifelike information that violates Newtonian motion (Mandler, 2012).

As a suggestion, the innate or at least predetermined sensitivity to animacy and life-like movements could be understood as a principle of saliency of the *ongoing changes within a multifactorial relational system,* including variations in relative speed, directions, and/or relative distance. From this perspective, for example, the natural behavior of physically constrained, form-invariant objects, or even mutually independent moving agents, could be seen as a frame of reference, a *neutral level from which non-inertial living forces or social configurations deviate.* In this way, animacy-related events are hardly predictable and become more visually salient, thus attracting the viewer’s attention. If this framework is appropriate, then a plausible working hypothesis would be that the more the kinematic changes are sudden, unpredictable, and incongruent with their neutral frame of reference, the more they will evoke impressions of animacy and agency (Tremoulet and Feldman, 2000; Lemaire et al., 2022).

More work is needed to further explore the empirical and theoretical plausibility of a life-detector rooted in the visual system and capable of capturing and integrating all of these relationships, as well as the interaction between perceptual animacy and the acquired knowledge about causal mechanisms (Schlottmann et al., 2006; Simion et al., 2011). Also, the relationship between appearance-based properties of animated objects and pure kinematics animacy-related constraints needs to be better explored in future research (Long et al., 2018; Spriet et al., 2022). For example, it is still unknown how kinematic-based animacy interacts with face-like invariants, and how both interact with other appearance-based animacy features, such as human and animal bodies. Furthermore, if lifelike kinematics easily and automatically induces the perception of animacy in newborns babies, might not the *absence of motion* in a static image itself act as a suppressing factor in the detection of animacy?

## Conclusion

As outlined in this review, the perceptual system seems to be extremely sensitive to the entire range of information conveyed by movement variations and interactions concerning living entities. From a phenomenological point of view, these kinetic configurations are widely evaluated as vivid and meaningful, in an unreflective way, independently from any prior knowledge about the objective nature of the stimuli or any inferential reasoning (Schlottmann and Shanks, 1992). The observer cannot help but see animacy and intentionality as attributes of the objects, even though he knows that it is not the case; the evident inert nature of the moving objects does not hamper this evocative, and quite powerful mechanism, as already noted by Michotte (1950/1991).

Additionally, with respect to the animacy-related visual properties - such as faces or bodies - visual sensitivity to movements brings the relevant advantage of detecting the presence of a living organism even when the visibility conditions are deficient, for instance when the moving object is far, dimly lit, out of focus, partially occluded or camouflaged, or simply unseen before. Even in these conditions, the simple kinetic structure of an event enable us to perceive the nature of the animate or of the social situation.

Many experimental findings have shown that animacy and intentional relationships between moving objects are extracted rapidly and automatically, are sensitive to subtle visual parameters, appear early in development, are present in non-human species and can interact with low level properties - such as visual performance, visual memory, and speed perception.

Moreover, animacy impressions elicited by kinematics and appearance-based animacy features appear dissociate and partially independent from each other (Simion et al., 2011). Observers such as human and non-human newborns - even other vertebrates such as chicks -, are sensitive and pay more attention to lifelike moving objects or patterns (i.e., point-light displays) than to inanimate events, regardless of form. In contrast, it appears that the development of appearance-based visual features associated with animate beings, with the exception of face-like invariants, is not present at birth and requires a period of learning.

In adulthood, this independency between kinematic constraints and appearance-based features still persists and allows a quite interesting side effect, as events might appear ambiguous but also aesthetically rich. In some everyday situations, we can see lifeless objects mimicking living creatures through lifelike movements. Thanks to the autonomy of the impressions of animacy induced by the pure kinematics, many natural events appear to be vitalized and “stop-motion” movies can animate and psychologize geometric shapes and other non-anthropomorphic objects. Actually, incongruity and paradoxicality are important ingredients of visual comedy Bergson, (1900/2013). For example, in Walt Disney’s classic movies (e.g., *Steamboat Willie,* 1928; *Fantasia,* 1940), as well as in many of Norman McLaren’s shorts (such as *A Chairy Tale,* 1957), co-directed by Norman McLaren and Claude Jutra for the National Film Board of Canada or even in many advertisements, animated agents behave like inanimate ones, and vice versa (see Thomas and Johnston, 1981; Ejzenstejn, 2004). Perhaps the fascinating character of these seemingly alive moving forms lies in this empirical evidence.

These elements support the hypothesis that we can visually shape high-level properties and that the visual system can directly perceive meaningful relationships and interactions between objects. In addition to the distinction between animate and inanimate, a general sensitivity to the ongoing changes in a multiple relational system from which non-inertial living forces or social configurations deviate has been proposed. This would make it possible to rapidly identify psychological, emotional, and social characteristics of lifelike kinematics.

In sum, kinematics appears to be a crucial cue of animacy and agency, even independently from other appearance-based properties. Living-like shaped visual objects can look alive only in virtue of their motion (at least that of breathing), vice versa if still they look dramatically life-less. On the contrary, inanimate life-like moving objects, even in contrast with their other visual features, can appear paradoxically alive.

By way of conclusion, the topic of animacy is rooted in Michotte’s experimental phenomenology, that is systematic psychophysical manipulation of stimuli configurations combined with subjective reports (see Costall, 2014; Bianchi and Davies, 2019; Parovel, 2019), and it is triggering a growing corpus of research cutting across several disciplines, including visual perception, developmental psychology, animal cognition, social psychology, cognitive neurosciences and robotics. Animacy, thus, besides being fascinating in itself, represents also a fruitful and challenging subject for empirical intersections and theoretical dialogue among different areas in experimental psychology. These dimensions of the visual scene, such as other expressive qualities of events that are still awaiting to be discovered, should be recognized and explored in all their richness and complexity within a multidisciplinary approach to human perception.

## Author contributions

GP conceived and wrote the entire manuscript.

## Funding

This publication was co-financed by POR CREO FESR Toscana 2014-2020 azione 1.1.5 sub-azione a1 – Bando 1 “Progetti Strategici di ricerca e sviluppo”: ATLAS.

## Conflict of interest

The author declares that the research was conducted in the absence of any commercial or financial relationships that could be construed as a potential conflict of interest.

## Publisher’s note

All claims expressed in this article are solely those of the authors and do not necessarily represent those of their affiliated organizations, or those of the publisher, the editors and the reviewers. Any product that may be evaluated in this article, or claim that may be made by its manufacturer, is not guaranteed or endorsed by the publisher.
